# The cross-pathway control system regulates production of the secondary metabolite toxin, sirodesmin PL, in the ascomycete, *Leptosphaeria maculans*

**DOI:** 10.1186/1471-2180-11-169

**Published:** 2011-07-26

**Authors:** Candace E Elliott, Ellen M Fox, Renee S Jarvis, Barbara J Howlett

**Affiliations:** 1 School of Botany, the University of Melbourne, Victoria, (3010), Australia; 2 Department of Sustainability and Environment, Gippsland Regional Office, (71 Hotham Street), Traralgon, Victoria (3844), Australia

## Abstract

**Background:**

Sirodesmin PL is a secondary metabolite toxin made by the ascomycetous plant pathogen, *Leptosphaeria maculans*. The sirodesmin biosynthetic genes are clustered in the genome. The key genes are a non-ribosomal peptide synthetase, *sirP*, and a pathway-specific transcription factor, *sirZ*. Little is known about regulation of sirodesmin production.

**Results:**

Genes involved in regulation of sirodesmin PL in *L. maculans *have been identified. Two hundred random insertional T-DNA mutants were screened with an antibacterial assay for ones producing low levels of sirodesmin PL. Three such mutants were isolated and each transcribed *sirZ *at very low levels. One of the affected genes had high sequence similarity to *Aspergillus fumigatus cpcA*, which regulates the cross-pathway control system in response to amino acid availability. This gene was silenced in *L. maculans *and the resultant mutant characterised. When amino acid starvation was artificially-induced by addition of 3-aminotriazole for 5 h, transcript levels of *sirP *and *sirZ *did not change in the wild type. In contrast, levels of *sirP *and *sirZ *transcripts increased in the silenced *cpcA *mutant. After prolonged amino acid starvation the silenced *cpcA *mutant produced much higher amounts of sirodesmin PL than the wild type.

**Conclusions:**

Production of sirodesmin PL in *L. maculans *is regulated by the cross pathway control gene, *cpcA*, either directly or indirectly via the pathway-specific transcription factor, *sirZ*.

## Background

Sirodesmin PL is the major phytotoxin produced by the plant pathogen *Leptosphaeria maculans *(Desm.), the causal agent of blackleg disease of *Brassica napus *(canola). Sirodesmin PL has antibacterial and antiviral properties [1] and is essential for full virulence of *L. maculans *on stems of *B. napus *[2]. This toxin is an epipolythiodioxopiperazine (ETP), a class of secondary metabolites characterised by the presence of a highly reactive disulphide-bridged dioxopiperazine ring synthesised from two amino acids (for review see [3]). The first committed step in the sirodesmin biosynthetic pathway is prenylation of tyrosine [4,5].

As for other fungal secondary metabolites, the genes for the biosynthesis of sirodesmin PL are clustered. The sirodesmin cluster contains 18 genes that are co-ordinately regulated with timing consistent with sirodesmin PL production. Disruption of one of these genes, *sirP*, which encodes a peptide synthetase, results in an isolate unable to produce sirodesmin PL [6]. Based on comparative genomics, the cluster of genes in *Aspergillus fumigatus *responsible for the biosynthesis of another ETP, gliotoxin, was then predicted. The pattern of expression of the clustered homologs was consistent with gliotoxin production [7]. The identity of this gene cluster was confirmed via the disruption of peptide synthetase, *gliP *whereby the resultant mutant was unable to make gliotoxin [8,9].

These ETP gene clusters also encode a Zn(II)_2_Cys_6 _transcription factor, namely SirZ for sirodesmin, and GliZ for gliotoxin [7]. Such factors are often found in biosynthetic gene clusters for secondary metabolites and they regulate transcription of the biosynthetic genes and consequently metabolite production. Disruption of *A. fumigatus gliZ *resulted in a mutant isolate unable to produce gliotoxin [10]. RNAi-mediated silencing of *sirZ *in *L. maculans *revealed that *sirZ *is essential for the transcription of sirodesmin biosynthetic genes and consequently production of sirodesmin PL [11]. In this paper we describe the identification of three genes that regulate sirodesmin PL and are unlinked to the sirodesmin gene cluster. One of these genes is denoted as *cpcA *(cross pathway control A), and is involved in regulation of amino acid biosynthesis in fungi such as *Saccharomyces cerevisiae, Aspergillus nidulans, and A. fumigatus *[12-14]. This pathway acts as a metabolic switch to enable the fungus to synthesize amino acids during periods of amino acid limitation. In this paper we describe the effect of starvation on the expression of sirodesmin biosynthetic genes and sirodesmin PL production in *L. maculans *wild type and *cpcA*-silenced isolates.

## Results

### Identification of genes flanked by T-DNA insertions in sirodesmin-deficient mutants of *L. maculans*

**Figure 1:**
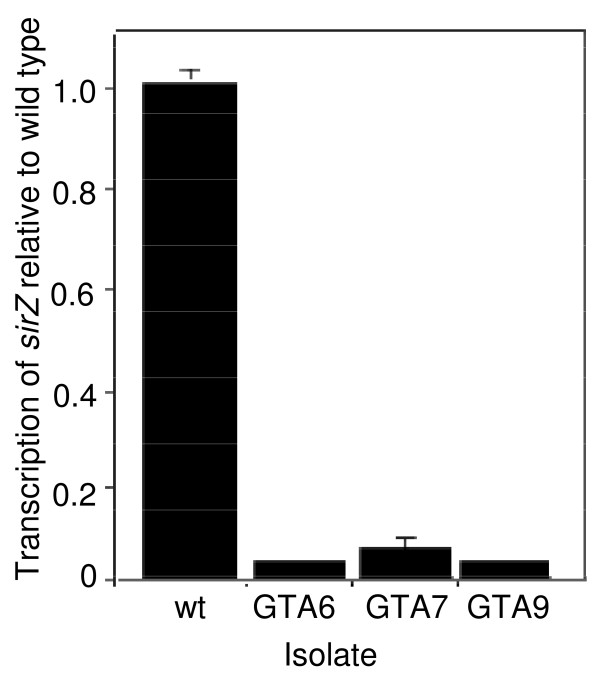
**Quantitative Reverse Transcription PCR analysis of the sirodesmin pathway-specific transcription factor, *sirZ*. in *Leptosphaeria maculans *wild type (IBCN 18) and sirodesmin-deficient mutants GTA6, GTA7 and GTA9**. Cultures were grown for six days in 10% V8 juice. Gene expression level is normalised to that of actin. Values are means ± SE of triplicate reactions of three independent biological samples. This experiment was repeated twice and consistent results were seen.

To generate sirodesmin-deficient mutants of *L. maculans*, wild type isolate IBCN 18 was transformed with plasmid pGTII, which contains T-DNA with a selectable marker (hygromycin-resistance) thus generating random insertional mutants [15]. Two hundred such mutants were then screened using a bioassay that exploits the antibacterial properties of sirodesmin PL [2]. Six-day-old cultures of the mutants grown on 10% Campbell's V8 juice agar were overlaid with a suspension of *Bacillus subtilis*. The presence or absence of zones of clearing of the bacterial lawn around the fungal colony 16 h later reflected the presence or absence, respectively, of sirodesmin PL. Three mutants, as well as a previously characterized mutant in the peptide synthetase gene (*ΔsirP*) in the sirodesmin biosynthetic pathway [6], did not clear the *B. subtilis *lawn. Sirodesmin-deficiency of these mutants was confirmed by HPLC analysis of filtrate of six-day-old cultures grown on 10% Campbell's V8 juice, whereby a peak eluting at 18.2 min in the wild type and co-incident with that of sirodesmin PL, was absent from profiles of the three mutants (data not shown). Quantitative RT-PCR showed extremely low levels of transcripts of the sirodesmin pathway-specific transcription factor, *sirZ*, in the three T-DNA mutants compared to the wild type strain (Figure 1). In these mutants a single copy of T-DNA had inserted in either the 5' or 3' untranslated regions of predicted genes (Table 1).

**Table 1:** Genes adjacent to T-DNA insertion in sirodesmin-deficient mutants of *Leptosphaeria maculans*

Mutant; Gene closest to T-DNA insertion, GenBank #	Site of T-DNA insertion in relation to coding region	Conserved domain	Best matches to NCBI database: Gene name (identifier), organism	GenBank #	E value
GTA6; *dsp1*; GU332622	315 bp downstream	Fungal specific DUF1752	hypothetical protein PTT_0874 *Pyrenophora teres f. teres 0-1*	EFQ94295.1	0
			PTRG_06770	XP_001937103	0
			*Pyrenophora tritici-repentis *Pt-1C-BFP		

GTA7; *dsp2 (cpcA); *GU332623	210 bp downstream	Basic region leucine zipper	hypothetical protein PTT_10495 *P. teres *f. teres *0-1*	EFQ92415.1	4e^-72^
			cross-pathway control protein 1 PTRG_00426	XP_001930759	1e-^70^
			*P. tritici-repentis*		

GTA9; *dsp3; *GU332624	209 bp upstream	Zn(II)2Cys6-DNA binding	predicted protein [*Aspergillus terreus *NIH2624]	XP_001209939	4e^-38^
			hypothetical protein AN5274.2	XP_662878	4e-^34^
			*A. nidulans*		

These genes were named *dsp *(deficient in sirodesmin production) and one of them (*dsp1 *in mutant GTA6) was predicted to encode a hypothetical protein with a fungal-specific domain (DUF1752) of unknown function. The closest match was to a hypothetical protein from the dothideomycete, *Pyrenophora teres *f *teres*. The other two genes, *dsp2 *and *dsp3 *(in mutants GTA7 and GTA9, respectively), encoded putative transcription factors; dsp3 had a Zn(II)_2_Cys_6 _DNA- binding domain, whilst dsp2 had a leucine zipper region. This latter transcription factor had best matches to a hypothetical protein from *P. teres *f *teres *and cross-pathway control protein 1 in P. *tritici-repentis *and also a significant match to CpcA in *Aspergillus fumigatus *(38% identity, 50% similarity). While the two *Pyrenophora *proteins were reciprocal best hits, CpcA of *L. maculans *was the next best match. This single copy *L. maculans *gene was denoted as *cpcA *and characterised further as described below.

### Preliminary analysis of *L. maculans cpcA*

**Figure 2:**
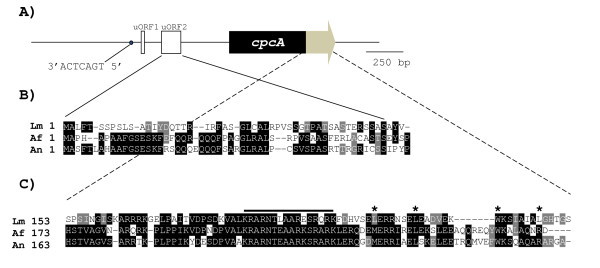
**A) The *cpcA *locus of *Leptosphaeria maculans***. The conserved leucine zipper region at the C-terminus of *cpcA *is dark grey. The open boxes indicate the upstream Open Reading Frames uORF1 and uORF2 in the 5' leader region. The black dot preceding them represents the putative cross-pathway control element (CPRE) whose sequence is 5'TGACTCA3'. **B) **Alignment of the deduced amino acid sequence of uORF2 with counterparts in the leader sequences of *Aspergillus fumigatus *(Af) *cpcA *(GenBank XP_751584.1) and *A. nidulans *(An) *cpcA *(GenBank AF302935). Black boxes with white text denote amino acids identical in two of the three fungal species. Grey boxes with white text mark conserved changes. Gaps are introduced to optimize alignment. **C) **Alignment of the deduced amino acid sequence of the C-terminus conserved leucine zipper region with that of *A. fumigatus *CpcA and *A. nidulans *CpcA. The thick black line denotes the bZIP transcription factors basic domain signature (PS00036). Asterisks mark positions where conserved leucine residues characteristic of a leucine zipper (L-x(6)-L-x(6)-L-x(6)-L) should be found.

Bioinformatic analysis revealed that *L. maculans cpcA *is intronless and encodes a predicted protein of 246 amino acids (Figure 2A). This finding was confirmed by PCR-amplification of either genomic DNA or cDNA with the wild type isolate as template. To characterize regions upstream of the *cpcA *transcript, 5' RACE was carried out. Similar to previously characterised *cpcA *homologs in *Aspergillus *sp. [13], two upstream open reading frames (uORFs) were identified at positions -541 bp (uORF1) and - 344 bp (uORF2), relative to the predicted first AUG of the *cpcA*-encoding region (Figure 2A). Upstream ORF1 was 12 bp, and encoded MAAI, whereas uORF2 was 159 bp and had high sequence similarity to uORF2 mapped in the 5' leader region of *A. fumigatus cpcA *and *A. nidulans cpcA *(Figure 2B). RACE analysis of the 3' untranslated region showed that the transcript from mutant GTA7 was truncated prematurely by the insertion of 36 bp from the left border of the T-DNA and lacked 127 bp of sequence upstream of the predicted polyadenylation site. The carboxy terminus of CpcA contained a region similar to the basic region of bZIP superfamily of transcription factors with strong sequence similarity to that of the homolog in *A. fumigatus *or *A. nidulans *(Figure 2C). In contrast with the *Aspergillus *homologs, the leucine zipper region contained three conserved leucine residues characteristic of a leucine zipper L-x(6)-L-x(6)-L-x(6)-L (Figure 2C). As expected for a protein with a transcription factor domain, CpcA was predicted by PSORT II to be localised in the nucleus (69.6% probability) and SignalP did not predict the presence of an N-terminal signal peptide (98.7% probability). In *A. nidulans, cpcA *transcription is autoregulated via cross pathway regulatory elements (CPRE) 5' TGA-(C/G)-TCA-3' in the *cpcA *promoter [13]. Point mutations in CPRE lead to low levels of *cpcA *transcripts and CpcA protein, when amino acids are limited. Such an element matching the consensus was present on the minus strand in the promoter region of *L. maculans cpcA *(-698 to -703).

### Role of CpcA in sirodesmin PL production in *L. maculans*

Although insertion of the T-DNA downstream of *cpcA *in mutant GTA7 reduced the transcript size by 127 bp, it did not reduce transcript levels of *cpcA *compared to those of the wild type (data not shown). Since the efficiency of gene disruption in *L. maculans *is very low, RNA mediated silencing was exploited to develop an isolate with extremely low levels of *cpcA *transcripts in order to study the effect of *cpcA *on sirodesmin PL production. Several putatively-silenced transformants were analysed and one, cpcA-sil, with 10% transcript level of that in wild type, as seen by q RT-PCR analysis, was chosen for further analysis (data not shown).

The effect of silencing *cpcA *on transcript levels of amino acid biosynthetic genes, sirodesmin biosynthetic genes, as well as the production of sirodesmin PL was then examined. The wild type and silenced isolate were grown for eight days in Tinline medium [16], which contains 83 mM glucose and 2 mM asparagine as carbon and nitrogen sources. Since starvation for at least one amino acid is sufficient to induce *cpcA *expression in *A. fumigatus *[14], amino acid starvation was induced in cultures of *L. maculans *wild type and cpcA-sil isolates by addition of the 'false feedback' inhibitor, 3-aminotriazole (3AT), a histidine analog that inhibits the histidine biosynthetic enzyme, imidazole glycerol phosphate dehydratase [17]. Five hours later, levels of transcripts of several genes relative to actin were measured by q RT-PCR.

**Figure 3:**
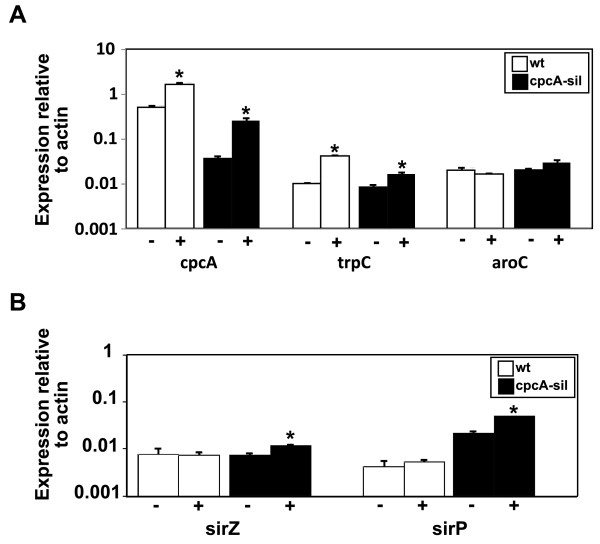
**Quantitative Reverse Transcription PCR analysis of (A) *cpcA, trpC *and *aroC*, (B) *sirZ *and *sirP *in wild type (wt) and a *cpcA*-silenced (cpcA-sil) isolate of *Leptosphaeria maculans***. Six replicates of each isolate were grown in Tinline for eight days and then mycelia were washed and then transferred to fresh Tinline media for 5 h with 5 mM 3AT (+) or without 3AT (-). RNA was isolated from all treatments, cDNA prepared and q RT-PCR carried out. Transcript level is normalised to that of actin. Values are means ± SE of triplicate reactions of three independent biological samples. Asterisks mark values that have a significant increase (p < 0.05) in mean transcription levels compared to controls without 3AT.

In the absence of 3AT, transcript levels of *cpcA *in the silenced isolate, cpcA-sil, were 7% of that of wild type. In the presence of 5 mM 3AT, transcript levels of *cpcA *increased significantly in the wild type (3 fold; p = 0.004) and in the silenced isolate (6 fold; p = 0.009) and yet the transcript levels of *cpcA *in the silenced isolate remained only 16% of that of wild type (Figure 3A). Next the ability of *L. maculans *CpcA to regulate amino acid biosynthesis was examined. In *Aspergillus *spp., transcript levels of tryptophan synthase, *trpC*, increase upon amino acid starvation, but remain low in isolates that are mutated in *cpcA*, whereas transcript levels of chorismate synthase, *aroC*, remain unchanged [14,18]. After 8 days in Tinline media, there was no significant difference in transcript levels of trpC of wild type or silenced isolates of *L. maculans *(data not shown). As expected, transcript levels of *trpC *increased significantly in wild type *L. maculans *in the presence of 5 mM 3AT (4 fold; p = 0.0003); a smaller increase was seen in the *cpcA-*silenced isolate (2 fold; p = 0.01). No significant differences in transcript levels of *aroC *were observed, even during amino acid starvation (Figure 3A). The levels of transcripts of *sirZ *and *sirP*, which are involved in sirodesmin PL biosynthesis did not differ significantly (p = 0.9 and 0.5) in the wild type in the presence or absence of 5 mM 3AT. However, there was a significant increase in transcript levels of *sirZ *(p = 0.008) and *sirP *(p = 0.0005) in the *cpcA*-silenced isolate after 5 h of amino acid starvation (Figure 3B).

**Figure 4:**
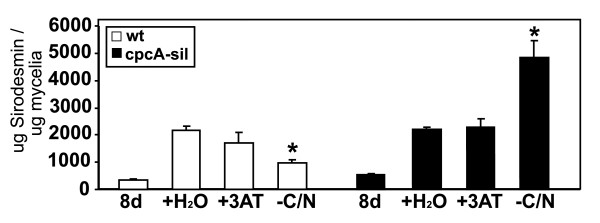
**Sirodesmin PL levels in culture filtrates of in wild type (wt) and a *cpcA*-silenced (cpcA-sil) isolate of *Leptosphaeria maculans***. Cultures were grown for eight days in Tinline media (8d) and the culture filtrate isolated and sirodesmin PL levels were quantified by HPLC. Mycelia were washed then transferred to fresh Tinline medium with water (+H_2_O) or 5 mM 3AT (+3AT), or Tinline medium with no carbon or nitrogen sources (-C/N) for a further eight days. Culture filtrates from the three treatments (+H_2_O, +3AT, -C/N) were extracted and sirodesmin PL levels were quantified by HPLC. Values are means ± SE of three independent biological samples. Asterisks mark values that have a significant increase or decrease (p < 0.05) in sirodesmin PL production compared to water controls (+H_2_O).

The effect of amino acid starvation on production of sirodesmin PL could not be determined in the experiments described above. Five hours of 3AT treatment would not be long enough to affect production of sirodesmin PL as this molecule is not detected until at least four days of growth in 10% V8 juice media [6]. Accordingly the wild type and *cpcA*-silenced isolate were grown for eight days on Tinline medium. Mycelia were washed and grown for a further eight days in Tinline, or Tinline with 5 mM 3AT or Tinline with no carbon or nitrogen (ie. lacking glucose and asparagine). Both isolates made low amounts of sirodesmin PL after the initial eight days of growth. After a further eight days, the amount of sirodesmin PL increased four to six fold in wild type and *cpcA*-silenced cultures, but there was no significant difference in the amount of sirodesmin PL, whether or not 3AT had been added to the cultures (Figure 4). However, in the absence of any carbon or nitrogen source (-C/N) there was half the amount of sirodesmin PL in wild type compared to cultures grown in the absence of 3AT (p = 0.003). In the *cpcA*-silenced mutant grown in the absence of any carbon or nitrogen source (-C/N) there was twice as much sirodesmin PL than in cultures grown in the presence or absence of 3AT (p = 0.05) (Figure 4).

## Discussion

Production of fungal secondary metabolites is often regulated by pathway-specific transcription factors, acting through global transcription factors that control several physiological processes and respond to environmental cues such as pH, temperature, and nutrition [19]. Given this complexity of regulation, it is not surprising that 1.5% of T-DNA insertional mutants of *L. maculans *analysed were sirodesmin-deficient. The finding that sirodesmin-deficiency correlated with severely reduced transcript levels of the pathway-specific transcription factor, *sirZ*, is consistent with studies on the regulation of production of other secondary metabolites. For instance, LaeA a master regulator of secondary metabolism in fungi such as *Aspergillus *spp. [20], regulates gliotoxin in *A. fumigatus *via the pathway-specific transcription factor, gliZ [10].

Cross pathway control homologs have a complex pattern of regulation. All identified to date are transcriptionally regulated in varying degrees; levels of transcripts increase significantly during amino acid starvation (for example, *S. cerevisiae Gcn4p *[12,21]. *N. crassa cpc1 *[22], *A. nidulans cpcA *[13], *A. fumigatus cpcA *[14] and *F. fujikuroi cpc1 *[23]). A CPRE element with one different nucleotide to that of the canonical CPRE sequence (5'-TGACTgA-3') is also present in the promoter of *sirZ *(-610 to -616), which suggests that CpcA may regulate *sirZ *directly. This element is not present in the promoter region of other genes in the sirodesmin gene cluster. Unfortunately due to the recalcitrance of *L. maculans *to homologous gene disruption we were unable to mutate the putative CPRE in the promoter of *sirZ *and test for regulation of sirodesmin PL production via CpcA.

The best studied cross pathway control homolog is *S. cerevisiae GCN4*. Starvation for any of at least 11 of the proteinogenic amino acids results in elevated transcript levels of targets of Gcn4p. Such targets include enzymes in every amino acid biosynthetic pathway, except that of cysteine, and also in genes encoding vitamin biosynthetic enzymes, peroxisomal proteins, mitochondrial carrier proteins, and autophagy proteins [12,21]. A comparative study of genes regulated by *S. cerevisiae *Gcn4p, *Candida albicans *CaGcn4p and *N. crassa *Cpc1 revealed regulation of at least 32 orthologous genes conserved amongst all three fungi [24]. These genes mainly comprised amino acid biosynthetic genes including the tryptophan biosynthetic gene *trpC *[13,14,22,25]. However, *aroC*, which encodes chorismate mutase, the enzyme at the first branch point of aromatic amino acid biosynthesis, is unresponsive to the cpc-system [14,18]. As expected, CpcA regulated transcription of *trpC *in *L. maculans *but not of *aroC *in response to amino acid starvation.

The cross pathway control system is also regulated at the translational level, since mutation of upstream uORFs in *A. nidulans *or *S. cerevisiae *results in increased translation of *cpcA *and *GCN4 *proteins under non-starvation conditions, compared to the wild type strains [13,26]. In *L. maculans *the *cpcA *coding region is preceded by two upstream Open Reading Frames (ORFs), the larger one displaying sequence similarity to an uORF preceding the coding region of *cpcA *of *A. fumigatus *and *A. nidulans*. Thus it is likely that *L. maculans cpcA *is regulated translationally, as well as transcriptionally.

It is puzzling why the insertion of T-DNA into the 3' UTR of *cpcA *in mutant GTA7 reduces production of sirodesmin PL but does not appreciably affect levels of *cpcA *transcript. One explanation is that the T-DNA insertion affects the regulation or increases the stability of the *cpcA *transcript, resulting in a cross pathway control system that is active in complete media and thus diverts amino acids from sirodesmin production. The importance of the 3' UTR in the regulation of genes is well-documented. For instance, regulatory elements in the 3' UTR control transcript stability of the global nitrogen regulator *AreA *in *A. nidulans *[27]. Deletions in 3' UTR of this gene render the transcript insensitive to nitrogen availability. Similarly, the deletion of part of the 3' UTR of *cpcA *could render the *L. maculans *isolate insensitive to amino acid levels in the media.

Given that sirodesmin PL is derived from two amino acids, tyrosine and serine, the finding that the transcription of sirodesmin biosynthetic genes, *sirP *and *sirZ*, and sirodesmin PL production appears to be regulated by *cpcA *and by amino acid starvation is not unexpected. It should be noted, however, that integration site effects may have contributed to these phenotypes since the site of insertion of the *cpcA*-silencing vector in the genome was not determined. It is unclear why the addition of 5 mM 3AT did not have as marked an effect as extreme starvation (absence of carbon and nitrogen) did on the levels of sirodesmin PL in either the wild type or *cpcA*-silenced isolate, when there was a marked effect on transcript levels of *sirP *and *sirZ *with addition of 3AT. This may be due to the significant difference in time periods during which the cultures were treated with 3AT; transcript levels were determined after 5 h, whilst sirodesmin PL levels were measured after eight days, after which time 3AT may have been depleted or degraded. In previous studies using 3AT to induce starvation, the effects on gene transcription were measured after 2 to 8 h [14,23,28]. Thus the imidazole glycerol phosphate dehydratase might have been inhibited for only a short period in the *L. maculans *cultures that were treated for eight days with 3AT. In the wild type culture grown in the absence of carbon and nitrogen, cross pathway control would be active during the entire eight days resulting in reduced levels of sirodesmin PL. In contrast, in the *cpcA*-silenced isolate grown in the absence of carbon and nitrogen, there is probably insufficient *cpcA *transcript to downregulate production of sirodesmin PL thereby resulting in an increased level of sirodesmin PL.

Until this report such a link between CpcA and secondary metabolism had only been implicated in two filamentous fungi. In *A. nidulans*, biosynthesis of penicillin is regulated by CpcA [28]. Penicillin and lysine share a common intermediate, the non-proteinogenic amino acid, α-aminoadipate. Under amino acid starvation conditions, CpcA directs metabolic flux towards lysine biosynthesis instead of penicillin biosynthesis, whilst in nutrient-rich conditions, penicillin is produced. In *F. fujikoroi*, *cpc1 *has been implicated in control of production of diterpenoid gibberellins, as deletion of glutamine synthetase leads to down regulation of gibberellin biosynthetic genes and upregulation of *cpc1 *[29]. However, recent experiments have shown that Cpc1 is not responsible for down-regulation of gibberellin biosynthesis [23].

Since *cpcA *regulates sirodesmin PL production, its homolog in *A. fumigatus *may regulate production of the related molecule, gliotoxin. An *A. fumigatus cpcA *mutant was attenuated for virulence in pulmonary aspergillosis of neutropenic mice, which had been immunosuppressed with cyclophosphamide and corticosteroids [14]. However, the effect on gliotoxin production was not tested. Several research groups have shown that gliotoxin is not a virulence factor in such neutropenic mice, but is a virulence factor in mice that have retained neutrophil function after immunosuppression by corticosteroids alone (for review see [30]). In a study of infection of immature dendritic cells by *A. fumigatus*, gliotoxin biosynthesis genes were downregulated over time. However, this could not be attributed to cross pathway control because *cpcA *was not differentially expressed [31].

The following model for regulation of sirodesmin PL production is consistent with all these data. When wild type *L. maculans *is grown on complete medium, the cross pathway control system is inactive, and amino acid biosynthesis does not occur (or occurs at a low level), but sirodesmin PL is produced. In contrast during starvation, amino acids are diverted from sirodesmin biosynthesis towards amino acid biosynthesis. This effect is mediated either directly or indirectly through the sirodesmin pathway-specific transcription factor, *sirZ. O*ther transcription factors including LaeA and *dsp3 *may also regulate sirodesmin PL production either directly or indirectly through *sirZ *as is the case for LaeA with *gliZ *and gliotoxin [10].

## Conclusions

Production of sirodesmin PL, a secondary metabolite derived from two amino acids, is regulated in *L. maculans *by amino acid availability via the cross pathway control gene, *cpcA*, either directly or indirectly via pathway-specific transcription factor, *sirZ*. Production of other classes of fungal secondary metabolites that are derived from amino acids, for example, siderophores, might also be regulated via this cross-pathway control system. As more genes encoding biosynthetic enzymes for such molecules are identified, this hypothesis can be tested.

## Methods

### Screening T-DNA mutants of *L. maculans *and identification of mutated genes

Two hundred T-DNA insertional mutants generated by transforming wild type *Leptosphaeria maculans *isolate IBCN 18 with plasmid pGTII [15] were screened for ones with low levels of sirodesmin PL [2]. Six-day-old cultures grown on 10% Campbell's V8 juice agar grown at 22°C with a 12 h/12 h light/dark cycle were overlaid with a suspension of *Bacillus subtilis *(NCTC 8236) in Luria Broth agar. Plates were then incubated at 37°C and the presence of zones of clearing around the fungal colony was assessed after 16 h. A sirodesmin-deficient mutant, *ΔsirP*, with a deletion in the peptide synthetase required for sirodesmin PL biosynthesis [6], was a negative control for sirodesmin PL production.

Three isolates (GTA6, GTA7 and GTA9) that did not inhibit bacterial growth were characterised further. Genomic DNA was prepared from mycelia, digested with an enzyme that cuts once within the T-DNA, and then subjected to Southern analysis (data not shown). This confirmed that a single copy of T-DNA had integrated into each mutant. Thermal asymmetric interlaced (TAIL)-PCR using the primers E, CE37, CE38, CE39, CE40, CE41, CE42 (Table 2) in various combinations was performed to isolate sequences flanking the T-DNA insertions in the mutants. These flanking regions were each cloned into plasmid pCR^®^2.1-TOPO (Invitrogen). The sequences of the resulting plasmids were compared to the draft genome sequence of *L. maculans *isolate JN3 (Genoscope and Unité de Recherche Génomique Info, France) and 10 kb regions flanking these DNA fragments were analysed by FGENESH for presence of ORFs. Putative genes were BLASTed against the NCBI database to identify best matches. The site of the T-DNA insertion in relation to the nearest open reading frame was then determined. Domains in these putative genes were sought using NCBI Conserved Domain Databases, SignalP 3.0, and subcellular location of proteins was predicted using PSORT II.

**Table 2:** Oligonucleotide primers

Primer name	Sequence (5' to 3')
E	AGWGNAGWANCAWAGG
CE37	GTGTAAAGCCTGGGGTGCCTAATGAGTG
CE38	AGCTAACTCACATTAATTGCGTTGCG
CE39	CGGGGAGAGGCGGTTTG
CE40	CCCTCGAGGCCTGCATATTATTTCTACTG
CE41	TGTTTGGGGCAGGCATGTTGA
CE42	TCAGAGACAGCCAGGAGAAATCA
RT1	GTCAACAACTCGCCTTCCAT
RT2	TTAGCTTGCGGCTGAAGATT
RT2A	TTGATTGACTCCACCTGGTG
RT3	GAGAAGTGGAAGAGCATCGC
RT4	TGTTCTTTGTAAGCGATGCG
RT5	TCATTTTGGTTTTCGTTTTGG
GTA7seq4	CTCGAGGCGGATGTAGAGAA
cpcAPROBEF	CCCTCGGGTCTTGAACAGT
GeneRacer5'	GCACGAGGACACUGACAUGGACUGA
GeneRacer5'-nested	GCTGTCAACGATACGCTACGTAACG
5'cpcA1	GCGCGGGGCAAGACTTGAGTT
5'cpcA2	CGAAAGGCGCGGAACGCTAGA
GeneRacer3'	CGCTACGTAACGGCATGACAGTG
GeneRacer3'-nested	ATTCGCCTCAGGACTTTGTG
cpcAQF3'	GTCAACAACTCGCCTTCCAT
cpcAQR3'	AGAGTTGCGACGCTCAAGTT
act1F	TTCCAGCTTGGAGAAGTCGT
act1R	CTGACATCGACATCGCACTT
sirZFA	CCAAAAGGAAGCAGGAACAA
sirZRA	GCCGAGTCTGTATCCGAATG
sirPF	TCACATGGTGAAATCGGCTA
sirPR	AATTCCCAACGCATCAACTC
aroCF	AACATTCGCTTCCAGCTCAT
aroCR	TACCCTGTCGATCCTCGCT
trpCF	CCGACTGTCTCGAAGTCACA
trpCR	GCTTTTGCGTAGGTTCTTGC
sirZ2F	CCGAATTTCCCTTCAGTCAA
sirZ1R	CAATGGGTCTGGAATACGCT
cpcAPROBER	CATCGCTATTGCTCTCGGAC
cpcARNAiF	GGGGACAAGTTTGTACAAAAAAGCAGGCTTCATCAGACACCATGGCACT
cpcARNAiR	GGGGACCACTTTGTACAAGAAAGCTGGGTGGCTCCATGGACTGGCTACTG

Transcript levels of *sirZ *and of *cpcA*, normalised to those of *L. maculans *actin in the wild type isolate and the three T-DNA mutants were examined. RNA was prepared using the TRIzol reagent (Invitrogen) from mycelia of the wild type (IBCN 18) and the T-DNA mutants, which had been grown on 10% V8 juice. The RNA was DNaseI-treated (Invitrogen) prior to oligo (dT)-primed reverse transcription with SuperScript III (Invitrogen). Reverse Transcription-PCR (qRT-PCR) was carried out as described [6] using primers sirZFA and sirZFR (for *sirZ*), cpcAQPCRF and cpcAQPCR (for *cpcA*), and act1F and act1R (for actin).

### Characterisation of *L. maculans cpcA*

The mutated gene in GTA7 had a close match to *A. fumigatus cpcA*, which has been well-characterised, and is henceforth named *L. maculans cpcA*. Untranslated regions (UTRs) 5' and 3' of the transcript and the positions of exons and introns were identified as follows. Segments of cDNA corresponding to the *cpcA *transcript were amplified (primers RT1, RT2, RT2A, RT3, RT4, RT5, GTA7seq4 and cpcAPROBEF) and cloned into plasmid pCR^®^2.1-TOPO (Invitrogen) and sequenced. Rapid amplification of 5' and 3' cDNA ends (RACE) using a GeneRacer kit (Invitrogen) was performed. Libraries were generated from cDNAs of isolates IBCN 18 and GTA7. Sequences at the 5' end of *cpcA *were amplified using primers GeneRacer5' and GeneRacer5'-nested and gene-specific primers 5'cpcA1 and 5'cpcA2. Sequences at the 3' end of *cpcA *were amplified using GeneRacer primers GeneRacer3' and GeneRacer3'-nested and gene-specific primers cpcAPROBEF and GTA7seq4. Products were cloned into pCR^®^2.1-TOPO and sequenced.

### RNAi-mediated silencing of *L. maculans cpcA*

RNA mediated silencing was exploited to develop an isolate with low *cpcA *transcript levels. A silencing vector was developed as described by Fox *et al .*[11] and a 815 bp region was amplified from genomic DNA of isolate IBCN 18 using *attB1 *and *attB2 *tailed primers, cpcARNAiF and cpcARNAiR, respectively. This fragment was cloned into Gateway^® ^plasmid pDONR207 using BP clonase (Invitrogen) to create plasmid pDONRcpcA. The fragment was then moved from pDONRcpcA into plasmid pHYGGS in two opposing orientations using LR Clonase (Invitrogen) to create the *cpcA *gene-silencing plasmid, pcpcARNAi. This plasmid was transformed into isolate IBCN 18 and two hygromycin-resistant transformants were further analysed. They both contained a single copy of plasmid pcpcARNAi at a site remote from the native *cpcA *locus, as determined by Southern analysis (data not shown) and the one transformant, cpcA-sil, with the greatest degree of silencing of *cpcA *(90%) was used in this study.

### Transcriptional analyses

To examine transcript levels, *L. maculans *conidia (10^6^) of the wild type, IBCN 18, and of the silenced isolate, cpcA-sil, were inoculated into Tinline medium [16] (50 mL) in a petri dish (15 cm diameter) and grown in the dark, without agitation. After eight days, mycelia were filtered through sterile miracloth and washed in Tinline medium. A sample was harvested for transcript analysis. Triplicate samples of mycelia were transferred to the fresh media, which was supplemented with H_2_O or 5 mM of 3-aminotriazole (3AT) (Sigma), which induces amino acid starvation. After 5 h RNA was extracted from mycelia. The relative abundances of *cpcA, aroC, trpC*, *sirZ *and *sirP *were compared by quantitative RT-PCR using primer pairs; trpCF and trpCR (for *trpC*); aroCF and aroCR (for *aroC*), and sirPF and sirPR (for *sirP*), as well as primers for *cpcA *and *sirZ *as described above. In all these experiments transcript levels were normalized to those of *L. maculans *actin by quantitative RT-PCR using the SensiMix (dT) master mix (Quantace). Each bar on the graph represents the mean transcript level of biological triplicates with error bars representing the standard error of the mean. A student's T- test was used to determine whether differences in levels of transcripts between treatments were significant.

### Extraction and analysis of sirodesmin PL

For initial characterisation of sirodesmin PL content, the wild-type (IBCN 18), the three T-DNA mutants and the *cpcA-*silenced mutant were grown in still cultures of 10% V8 juice (30 ml) for six days. In experiments to determine the effect of amino acid starvation on sirodesmin PL production, triplicate cultures of the wild-type isolate and the *cpcA-*silenced mutant were grown in Tinline medium (30 ml). After eight days mycelia were filtered through sterile Miracloth, washed and transferred to 30 ml of fresh Tinline medium, or Tinline supplemented with 5 mM 3AT, or Tinline without any carbon or nitrogen-containing molecules. After a further eight days, mycelia were filtered through sterile Miracloth, freeze-dried and then weighed. Aliquots (5 ml) of culture filtrates were extracted twice with ethyl acetate. Production of sirodesmin PL was quantified via Reverse Phase-HPLC as described by Gardiner *et al .*[6]. A student's T- test was used to determine whether differences in levels of sirodesmin PL between treatments were significant.

## Competing interests

The authors declare that they have no competing interests.

## Authors' contributions

CEE developed the T-DNA insertional mutants, carried out quantitative RT-PCR analyses and quantified sirodesmin PL. EMF screened the T-DNA insertional mutants, undertook preliminary analyses of them and generated an *L. maculans *isolate silenced in *cpcA*. RJ quantified sirodesmin PL. BJH conceived the study, and drafted the manuscript. All authors read and approved the final manuscript.
